# Electronic Surveillance System for the Early Notification of Community-Based Epidemics (ESSENCE): Overview, Components, and Public Health Applications

**DOI:** 10.2196/26303

**Published:** 2021-06-21

**Authors:** Howard Burkom, Wayne Loschen, Richard Wojcik, Rekha Holtry, Monika Punjabi, Martina Siwek, Sheri Lewis

**Affiliations:** 1 Johns Hopkins University Applied Physics Laboratory Laurel, MD United States

**Keywords:** health surveillance, outbreak detection, population health

## Abstract

**Background:**

The Electronic Surveillance System for the Early Notification of Community-Based Epidemics (ESSENCE) is a secure web-based tool that enables health care practitioners to monitor health indicators of public health importance for the detection and tracking of disease outbreaks, consequences of severe weather, and other events of concern. The ESSENCE concept began in an internally funded project at the Johns Hopkins University Applied Physics Laboratory, advanced with funding from the State of Maryland, and broadened in 1999 as a collaboration with the Walter Reed Army Institute for Research. Versions of the system have been further developed by Johns Hopkins University Applied Physics Laboratory in multiple military and civilian programs for the timely detection and tracking of health threats.

**Objective:**

This study aims to describe the components and development of a biosurveillance system increasingly coordinating all-hazards health surveillance and infectious disease monitoring among large and small health departments, to list the key features and lessons learned in the growth of this system, and to describe the range of initiatives and accomplishments of local epidemiologists using it.

**Methods:**

The features of ESSENCE include spatial and temporal statistical alerting, custom querying, user-defined alert notifications, geographical mapping, remote data capture, and event communications. To expedite visualization, configurable and interactive modes of data stratification and filtering, graphical and tabular customization, user preference management, and sharing features allow users to query data and view geographic representations, time series and data details pages, and reports. These features allow ESSENCE users to gather and organize the resulting wealth of information into a coherent view of population health status and communicate findings among users.

**Results:**

The resulting broad utility, applicability, and adaptability of this system led to the adoption of ESSENCE by the Centers for Disease Control and Prevention, numerous state and local health departments, and the Department of Defense, both nationally and globally. The open-source version of Suite for Automated Global Electronic bioSurveillance is available for global, resource-limited settings. Resourceful users of the US National Syndromic Surveillance Program ESSENCE have applied it to the surveillance of infectious diseases, severe weather and natural disaster events, mass gatherings, chronic diseases and mental health, and injury and substance abuse.

**Conclusions:**

With emerging high-consequence communicable diseases and other health conditions, the continued user requirement–driven enhancements of ESSENCE demonstrate an adaptable disease surveillance capability focused on the everyday needs of public health. The challenge of a live system for widely distributed users with multiple different data sources and high throughput requirements has driven a novel, evolving architecture design.

## Introduction

### Background

In recent decades, public health disease surveillance relied on laboratory confirmation and passive participation. Often, the lack of automated detection and reporting resulted in time delays that impeded prompt mitigation activities. Public health institutions thus began using enhanced surveillance techniques with the potential for timely epidemic detection and tracking. These techniques have been incorporated in electronic and increasingly internet-based health surveillance systems for everyday use by health monitors. Intensive efforts to establish health surveillance systems occurred at multiple institutions and government agencies in the late 1990s. As a result, a substantial collection of review papers, system-specific evaluations, and evaluation criteria have emerged [1-4].

The Electronic Surveillance System for the Early Notification of Community-Based Epidemics (ESSENCE) system originated as a collaboration between two such projects. A project of Dr Michael Lewis, then a medical resident under Dr Julie Pavlin at the Walter Reed Army Institute for Research (WRAIR), applied US military clinic visit data for outbreak detection in a project called * ESSENCE* [5]. Concurrently, for the same purpose, Johns Hopkins University Applied Physics Laboratory (JHU/APL) combined civilian data from hospital emergency departments (EDs), physician office visits, over-the-counter (OTC) sales, and school absenteeism records, first with internal funding, then for the State of Maryland [6]. The two groups joined forces in anticipation of possible bioterrorist activity at the turn of the century as January 1, 2000, approached, and this collaboration, along with similar efforts at other institutions, led to further funded development in the multicenter Bio-Event Advanced Leading Indicator Recognition Technology (BioALIRT) program of 2001-2003 for the Defense Advanced Research Project Agency [7]. In leading one of four BioALIRT research teams, the JHU/APL and WRAIR groups further matured the ESSENCE system concept.

Following the terror attacks of September 11, 2001, ESSENCE was expanded in Maryland, increasingly operationalized in state and local civilian health monitoring agencies and implemented in all global US military treatment facilities. Since then, fueled by a succession of initiatives driven by both bioterrorism and natural public health concerns, versions of ESSENCE have been implemented in the Department of Defense (DoD), the Veterans Administration (VA), the Centers for Disease Control and Prevention (CDC), and state and regional public health agencies across the United States and, through collaborative DoD efforts, internationally. Following the widespread use of ESSENCE and enhancements at JHU/APL to meet users’ evolving needs, the CDC National Syndromic Surveillance Program (NSSP) adopted ESSENCE in 2014 as the standard analytic surveillance and visualization engine on the BioSense Platform for state and local public health monitors [8,9]. Nearly 6000 health care facilities covering 49 states and the District of Columbia contribute ED data to the BioSense Platform daily [10]. The user benefits of ESSENCE have long surpassed the *Early Notification* part of its acronym to include aspects of situational awareness, such as tracking and characterization of known health events, assessing the burden of all-hazard threats such as severe weather, environmental hazards, and substance abuse, and rumor control to enable improved public health response. In 2021, there are approximately 3800 ESSENCE users, including approximately 3000 recent frequent users. These users are spread among ESSENCE installations in 23 states and local jurisdictions, the DoD system, the NSSP system serving most US states, and several instances in private organizations.

A timeline detailing the historical development of ESSENCE is presented in Multimedia Appendix 1.

### Objectives

This study aims to describe how the architecture, analytics, visualizations, and user collaboration tools in ESSENCE have empowered public health at local and regional levels despite resources that are often scarce compared with those of other government institutions, with discussion of the lessons learned over the past 20 years and the continuing challenges of emerging population health threats. Through the main text and supplementary files on the technical details, this paper will describe the various ways by which ESSENCE enables user institutions to meet the standards set for public health surveillance systems [11], from engaging stakeholders to providing technical justification to sharing lessons learned. In the context of this paper, the *Methods* section refers to the data architecture, analytic methods, and visualizations provided by ESSENCE, and the *Results* section refers to the surveillance capabilities that have thus been realized by system users.

## Methods

This section describes the general functionality and main components of ESSENCE, including data types and management, analytic methods, and visualizations. The details of these components are provided in Multimedia Appendices 1-4.

### System Overview

ESSENCE is a health surveillance system that uses advanced analytics and visualizations to expose anomalies in both traditional and nontraditional public health data, with the goal of enabling public health to find and monitor outbreaks of health events and make decisions. ESSENCE users access a secure web-based tool to conduct disease surveillance for the purpose of timely detection, situational awareness, and descriptive epidemiologic analysis of baseline disease patterns and outbreaks. For an effective public health response, public health authorities must have the ability to identify the infected population so that further spread can be contained. Leveraging the near real-time availability of an increasing number of data sources, ESSENCE analytical and alerting capabilities provide an opportunity for public health users to capture the early stages of an outbreak and track its progress. ESSENCE enables the integration of electronic data from both clinical and nonclinical sources to enhance situational awareness. For most users, the primary clinical data source is hospital ED chief complaint records. In addition, based on availability, public health agencies incorporate other data types, such as OTC medication sales, poison control call center data, prescription drug data, reportable disease data, vital statistics mortality data, and school absentee data. Once raw data reach ESSENCE, the analysis, visualization, and communication features of ESSENCE allow the end user to gather and organize the resulting wealth of information into a coherent view of population health status and to communicate findings among other users and stakeholders.

As a result of ongoing user feedback daily and event-based disease surveillance needs, ESSENCE features include spatial and temporal statistical alerting, custom querying, user-defined alert notifications, geographical mapping, remote data capture, and event communications.

ESSENCE provides three main functions:

1. Data ingestion or preprocessing: traditional and nontraditional data sources are electronically received by ESSENCE, and many are mapped to syndrome groupings. During the ingestion process, data are cleansed (eg, duplicate records removed and invalid characters removed), updated (eg, existing records information is updated with new information), and categorized (eg, syndrome and subsyndrome categories assigned to records).
2. Alerting: multiple temporal and spatial alerting algorithms are applied to each data set to develop a list of alerts or flags for further investigation by public health officials. In addition to algorithms developed by JHU/APL and the WRAIR, ESSENCE can incorporate algorithms required by the jurisdiction where the system is deployed.
3. Analysis and visualization: ESSENCE data and alerts can be analyzed and visualized in multiple ways in the system, both spatially and temporally.

**Figure 1:**
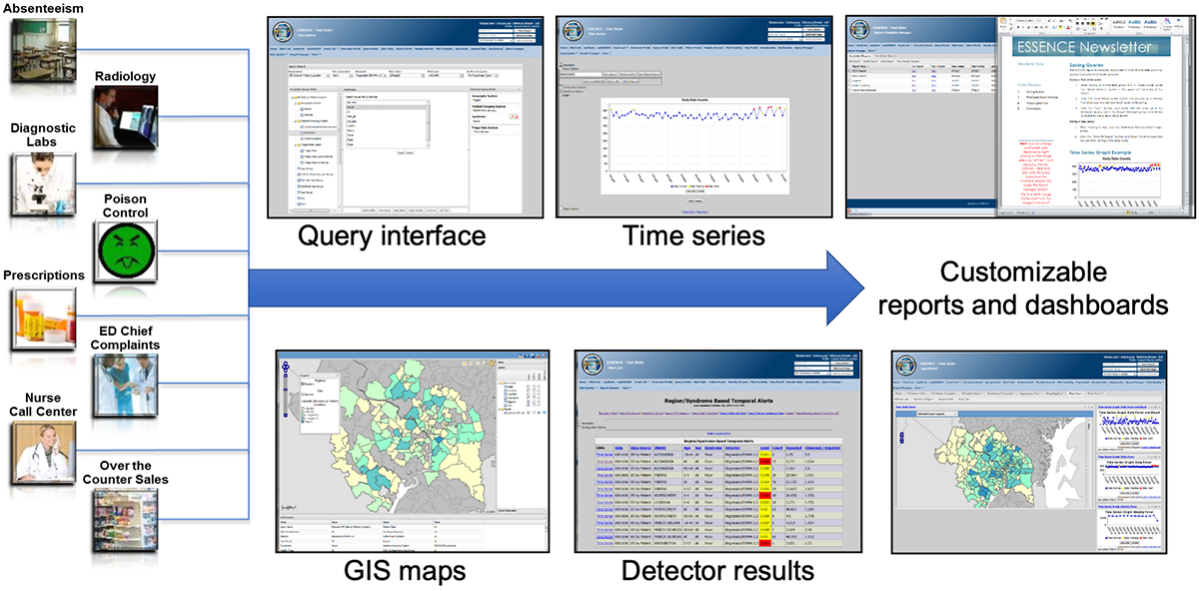
Schematic overview of Electronic Surveillance System for the Early Notification of Community-Based Epidemics (ESSENCE) features showing a sampling of users’ data types, analysis modes, and screen interfaces. ED: emergency department; GIS: geographic information system.

These component functions are illustrated schematically in Figure 1. They are introduced in the following sections, with an additional file for each to provide details.

### Data Types and Syndrome or Feature Selection

The types of data analyzed in ESSENCE are the prerogative and responsibility of the jurisdiction, although JHU/APL provides the capability for basic types. The ESSENCE system is data agnostic—the only requirement for a monitored data type is the inclusion of a data field. The data time resolution is also unrestricted. Data frequencies in ESSENCE range from seconds to years, although daily data are the most common. All but a few users monitor hospital ED data. Most ESSENCE user jurisdictions face the burden of acquiring their data sources, gaining approval for their routine intended use, and extracting features for monitoring. The level of effort varies greatly depending on the data source. Various users also monitor or have monitored the records of the data sources listed below. Sources known to be currently or formerly tracked among users’ ESSENCE systems for routine prospective monitoring include OTC remedy sales, physician office visits, laboratory test orders and results, school absenteeism and health office or nurse records, reportable disease cases, poison center call records, Assistant Secretary for Preparedness and Response Disaster Medical Assistance Teams data for disaster response [12], cardiovascular and other chronic diseases from inpatient encounters, livestock and companion animal health encounters, human vitals measurements, death records, emergency medical services and 911 calls, and climate and air quality data.

Users have also incorporated or adapted data from other surveillance systems, such as the National Poison Data System [13]. Although not all these sources have proven useful for routine surveillance, users have used the system to investigate their utility and to seek the best data usage.

In separate studies or projects, individual user jurisdictions or their research partners have also used ESSENCE to analyze records of radiology impressions; genomic sequencing data; zoo animal health; environmental sensor outputs; sales of specific products, such as thermometers, orange juice, and tissues; social media posts and searches; and fantasy sports data. Multiple content formats used in ESSENCE include Health Level-7 (HL-7) formats for hospital data and National Emergency Medical Services Information System (NEMSIS) formats for emergency medical services data. File formats include delimited or tabbed or fixed-width American Standard Code for Information Interchange (ASCII) text, XML, and JavaScript Object Notation (JSON).

Each data source has its own challenges for user jurisdictions to obtain sustained electronic access from data providers and any requisite government approval. When a data stream of any of these sources is acquired for routine monitoring, an immediate question is how to use the streaming data to track the health outcomes of interest. An often-applied procedure is to track the counts of subcategories of the data expected to correspond to these outcomes. These subcategories are commonly called *syndromes*, generalizing the medical definition of this term, denoting disease-related collections of signs and symptoms. Thus, in the surveillance context, a *syndrome* may refer to grouped hospital visits associated with a fixed collection of symptoms, laboratory tests ordered for certain conditions, web searches containing sets of terms, billing records covering any of a class of remedies, or other subgroups depending on the data source. Syndrome formation is a critical step that may use only a fraction of all streaming data and may produce few or many groups to track. The number and composition of syndromes depend on the richness of the data, the number of outcomes of interest, and the resources of the monitoring institution for investigation and response.

Syndromes and subsyndromes used in ESSENCE vary depending on the available clinical grouping systems and the needs of the user site. Early versions of ESSENCE formed syndrome groups using diagnosis codes, which have disadvantages of late assignment and emphasis on billing practice in many medical systems. Examples include respiratory and gastrointestinal subsyndromes, such as asthma. Categorization soon switched to the use of free-text chief complaint or reason-for-visit data fields. For this categorization, the JHU/APL ESSENCE team developed the Chief Complaint Processor (CCP), a versatile, stand-alone program for weighted keyword-based classification by free-text fields. The CCP is highly configurable, with tables including sets of syndromes and subsyndromes with classification rules allowing complex logic, positive and negative weighting of component terms, abbreviation and spelling rules, and a list of unmodifiable terms. For example, CCP puts a record with a chief complaint of *nausea* or *vomiting* in the gastrointestinal category. The CCP creates a ChiefComplaintsParsed field for the use of classification rules after treatment of abbreviations, some misspellings, and other cleanup [14]. These classifications have enabled additional natural language processing and machine learning initiatives by both ESSENCE developers and users, and findings from these initiatives are shared among users with each emerging health threat [15-17].

As with diagnosis code-based processing, syndrome groups are tabulated, plotted, and monitored each day with statistical alerting algorithms for early potential outbreak indications.

### Architecture, Security, Preprocessing, and Quality Management

**Figure 2:**
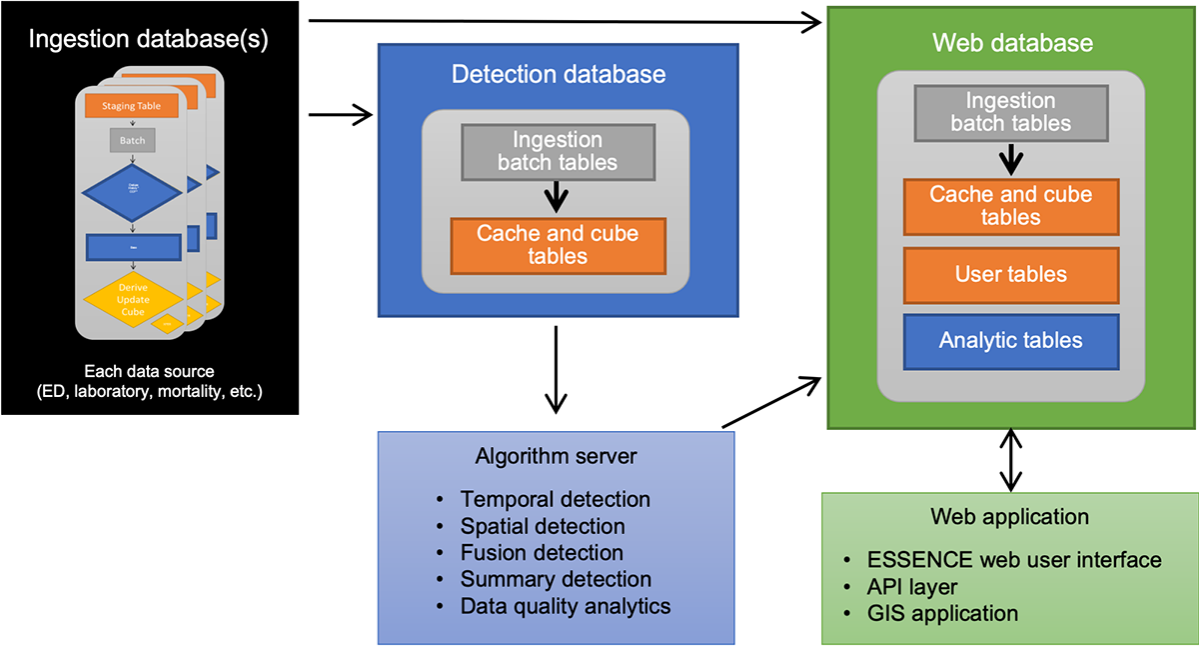
Flow diagram illustrating typical Electronic Surveillance System for the Early Notification of Community-Based Epidemics (ESSENCE) data flow with databases and tables, generalized to account for diversity among user needs and server configurations. API: application program interface; ED: emergency department; GIS: geographic information system.

The software architecture used for ESSENCE is a 3-tier web application with a presentation layer as a user front end, a business layer for the application of algorithms, and a back end for databases. All automated data transfers occur over secure virtual private networks. Multiple, data-dependent preprocessing steps include deduplication procedures, formation of syndrome fields, calculation of distances, and derivation of additional fields and flags based on jurisdictional business rules and logic. The flow diagram is shown in Figure 2. Multimedia Appendix 2 describes these features in detail.

### Alerting Methods Using Statistics and Artificial Intelligence

The following principles were derived from ESSENCE users to guide method selection and to clarify the interpretation of results.

General considerations include:

- These methods are not intended to identify outbreaks positively without supporting evidence. Their purpose is to direct the attention of limited monitoring staff with increasingly complex data streams to data features that merit further investigation. They have also been useful for corroborating clinical suspicions, rumor control, tracking of known or suspected outbreaks, monitoring of special events and health effects of severe weather, and other locally important aspects of situational awareness. Successful users value these methods more for the latter purposes and do not base public health responses solely on algorithm alerts.
- These algorithms are one-sided tests that monitor only unusually high counts, not low ones. Low counts could result from a critical outbreak situation that prevents data reporting, but there are many more common reasons for low counts (such as unscheduled closings or system problems), so the algorithms do not test for abnormally low counts.
- In addition to the data- and disease-specific considerations below, the algorithm selection was also driven by system considerations. Users need to monitor many types of data rapidly. External covariates, such as climate data or clinic schedules, may not be available for prompt analysis. Many methods in the literature, armed with retrospective data of a certain type, depend on the analysis of substantial history. Day-to-day users, often with only a small fraction of time available for monitoring, will not wait for several minutes for each query. Building separate algorithms for each user data stream is impractical, especially because sufficient data history is unavailable for many standard time series and all ad hoc series; therefore, ESSENCE methods have been adapted from the literature and engineered for appropriate matching to the data and timely results.
- If the time series monitored by algorithms represent many combinations of clinical groupings, age groups, and geographic regions, excessive alerting may occur simply because of the number of tests applied. The summary alert method was implemented to limit excessive alerting. This method is based on the control of the false discovery rate, that is, the expected ratio of false alerts to the total alert count, and its statistical implementation in ESSENCE is detailed in Multimedia Appendix 3. Aside from analytic methods to control alerting, default alert lists should be limited to results from the time series of interest to the user, either by system design or by the user’s active specification. For example, one method of reducing the default alert list is to restrict algorithms to all-age time series groupings. Depending on the scope of the user’s responsibility, the alert list may also be restricted according to both epidemiological interest and the resources available for investigation. For example, a monitor of a national-level system with algorithms applied to many facilities may be interested only in alerts with at least 5 to 10 cases. In circumstances of heightened concern, these restrictions can be relaxed, or the user can use ESSENCE advanced querying methods to apply algorithms to age groups and/or subsyndromes.

Individual alerting algorithms implemented in ESSENCE are listed and described in Multimedia Appendix 3.

### Visualization

Both standard and user-customizable visualizations are available in ESSENCE, many shaped in response to user ideas and requests. Highly configurable and interactive modes of data stratification and filtering, graphical and tabular customization, user preference management, and sharing features allow users to query data and view geographic representations, time series and data details pages, and reports. Figure 2 shows examples of the standard plots and maps. Multimedia Appendix 4 (Figures S1-S4) summarizes key features with explanations and screenshots of novel visualizations developed for ESSENCE to fulfill requests of the user community.

## Results

### Overview of Findings

The results in this section represent a broad range of ESSENCE applications, especially those not previously published in the peer-reviewed literature, which demonstrate how ESSENCE monitors health threats at national, state, and local jurisdictions. Rather than giving the results of one analytic method applied to a particular data set, we show the results of the data architecture, analytics, and visualizations described in the previous section for public health objectives achieved by users. The following sections demonstrate the system simplicity, flexibility, and acceptability, key attributes from published system evaluation guidelines [18]. The examples in these sections were chosen to show the breadth and depth of the application to meet these criteria. For the other attributes, each ESSENCE user has local surveillance objectives and constraints that impose the requirements of performance metrics, such as sensitivity, positive predictive value, and timeliness. Algorithms described in the analytic details supplement were developed to maximize the local sensitivity or positive predictive value tradeoff and other practical metrics.

In addition to the versions used and shared by the US surveillance community, the applications below refer to the open-source Suite for Automated Global Electronic bioSurveillance (SAGES) toolkit that ESSENCE developers have provided for non-US health monitors, including OpenESSENCE and ESSENCE Desktop editions [19,20]. On the basis of the features and functionality of ESSENCE, tools within SAGES were developed specifically for use in low- and middle-income countries with limited resources but do not include some of the more recent analytics and visualization tools requested by users across the NSSP.

### Infectious Disease Applications

Early applications of ESSENCE focused on the detection of infectious disease outbreaks, with much attention paid to influenza-like illness because it is a prodrome for multiple naturally occurring diseases and for many potentially weaponized for bioterrorism [21,22]. Civilian [23], military [22], and VA [24] ESSENCE users monitored for outbreaks of seasonal and nonseasonal outbreaks of febrile respiratory infections, gastrointestinal infections caused by contaminated food or water, and rarer infections.

Surveillance systems were particularly helpful in tracking the pandemic of the novel H1N1 influenza in 2009 [24]. The pandemic was important as an interregional use of surveillance systems to track a common threat. For example, the National Capital Region Disease Surveillance Network, comprising ESSENCE users at health departments in the District of Columbia and parts of the states of Maryland and Virginia, shares population-level disease incidence information to promote interjurisdictional surveillance. In 2009, this network allowed National Capital Region public health practitioners to track the course of the pandemic from the spring through the fall, comparing the overall and age-specific burden of illness to national and neighboring state trends. A broader collaboration between ESSENCE and non-ESSENCE system users adopted a standardized definition of influenza-like illness to enable uniform local tracking of the pandemic across the United States [25].

Users have applied ESSENCE to form and share queries for indications of other infectious diseases using text from chief complaints, discharge diagnosis, and triage notes when available. Infectious threats tracked in published examples include general waterborne diseases [26], tuberculosis [17], rabies [27], and Middle East Respiratory Syndrome [15]. In recent years, concerns over mosquito-borne diseases have generated new queries by multiple users and occasionally uncovered important cases [16].

The health department of Maricopa County, Arizona, presented an example of the benefits of surveillance systems in June 2018. The department had added an ESSENCE query for signs of Rocky Mountain spotted fever, which is not endemic to that county, because of concerns that cases transferred from endemic areas might be missed. A child’s patient record was signaled by the query, and the department contacted the hospital. This contact led to the reversal of a medication decision that might have been fatal to the child [28].

Such monitoring activities have repeatedly uncovered unreported cases of diseases for which reporting is mandatory [29]. These findings illustrate the importance of redundancy with systems such as ESSENCE to avoid missing important cases, even when traditional reporting mandates exist.

At the submission of this manuscript, the intense collaboration of ESSENCE users is focused on tracking the COVID-19 pandemic. General queries on COVID-like illness and specific ones involving pneumonia and specific symptom sets are being refined and shared [30].

### Applications for Tracking Burden of Severe Weather and Natural Disaster Events

Health departments have used ESSENCE for preparedness, health burden assessment, and response to severe storms and other natural disasters. The state of Oregon conducted a successful program to mitigate the effects of wildfires [31]. This program featured customized ESSENCE queries with other coordinated efforts among state and local health departments and preparedness teams. Among several states that use ESSENCE to monitor the effects of hurricanes, the Tennessee Health Department devised queries to determine the volume and clustering of patients in local hospitals because of storms in other states [32]. Effective monitoring of some events requires a combination of multiple data sources. Following a prolonged storm-related power outage, the health department of Seattle-King County, Washington, combined data from the ESSENCE ED data with ambulance calls and public utility data to monitor for cases and clusters of carbon monoxide poisoning and food poisoning [33]. More recently, the Florida Health Department monitored carbon monoxide poisoning after Hurricane Irma in 2017 [34]. Institutions using ESSENCE are increasingly incorporating environmental and other data sources in their systems for richer situational awareness of disaster-related health threats [35,36].

### Applications for Mass Gathering Surveillance

Scheduled mass gathering events such as political conventions and major athletic competitions concern population health monitors because (1) such events are bioterrorism opportunities to harm many victims and gain media attention, (2) infections through contaminated food or water could spread rapidly through the expanded population, (3) those visiting for several days could import infections or take them back to their own cities, and (4) a surge of patients could overwhelm local care provider resources. Adequate preparedness and response require coordination across jurisdictional boundaries, but privacy laws often restrict patient-level data sharing. ESSENCE syndrome definitions and queries have been customized for many such events. In 2007, Marion County, Indiana, and Cook County, Illinois, were home counties for the competing teams in Super Bowl XLI, and the game was hosted in Miami-Dade, Florida. The health departments of these geographically distant counties were ESSENCE users, and customization of their systems for the days surrounding the event helped coordinate surveillance despite only 2 weeks’ notice after the teams were determined [37].

A partnership between the Florida Department of Health and the US HHS Office of the Assistant Secretary for Preparedness and Response to improve the response of Disaster Medical Assistance Teams produced a new ESSENCE module that was deployed for health monitoring of the 2012 Republican National Convention in Tampa [36]. State and county health departments used ESSENCE for coordinated monitoring of crowds at the US Olympic Trials in July 2016 [38]. In January 2017, the Washington DC Department of Health used ESSENCE queries along with other data sources for health surveillance at the 58th US presidential inauguration [39].

For monitoring of events outside the United States, JHU/APL developed the SAGES system, the open-source version of ESSENCE designed for global resource-limited settings, in 2014 to monitor the 8th Micronesian Games held in Federated States of Micronesia, Pohnpei, and the 3rd International Conference on Small Island Developing States in Apia, Samoa [40].

### Applications for Chronic Disease and Mental Health Surveillance

The use of ESSENCE to monitor risk factors and incidence of chronic disease and mental health disorders has proliferated since a DoD ESSENCE study using clinic and prescription data to monitor behavioral health in 2004 [41]. The Boulder County, Colorado Health Department recently implemented and tested multiple queries to monitor mental health [42].

Addressing ESSENCE utility for chronic diseases in general, the Cook County Illinois health department applied machine learning methods to assess the utility of ESSENCE ED data for monitoring cardiovascular disease, acute myocardial infarction, acute coronary syndrome, stable angina, stroke, diabetes, hypertension, asthma, and chronic obstructive pulmonary disease. From correlational validation testing based on 8 full years of chief complaint text and electronic medical record data, they concluded that ESSENCE data are suitable for monitoring all these conditions except stable angina and hypertension “at local, state, or national levels” [43]. The Nebraska State Health Department has used ESSENCE to monitor for cardiovascular disease for several years, and the Florida State Department similarly monitors acute myocardial infarction incidence [44].

### Applications for Injury and Substance Abuse Surveillance

An unexpected but arguably the most helpful benefit of ESSENCE to health department users is to facilitate communication and collaboration among agency divisions. An important example in the context of the ongoing opioid overdose crisis has been the strengthening of connections between syndromic surveillance specialists and groups specializing in injury prevention, behavioral health, and drug abuse. Adaptations of ESSENCE included queries for both prescription and illicit drug types and overdose cluster detection to help inform public health response tactics, such as needle exchange and naloxone distribution.

Multiple health departments have applied ESSENCE to gain awareness of the locations and subpopulations at risk of injuries from falls [45]. The Boston Public Health Commission in Massachusetts used it to monitor for hearing loss, acute depression, and explosion-related injuries in the aftermath of the 2013 Boston Marathon bombing and subsequent manhunt. The St Louis Missouri Health Department established ESSENCE queries for injuries indicative of bomb-making activities [46].

Monitoring for substance abuse is common among ESSENCE users. The Tri-County Health Department in Colorado uses its system to investigate the adverse effects of marijuana use [47]. The Florida State Department queries for ED visits resulting from synthetic marijuana [48] and for novel street drugs, such as Flakka, as they become known public health problems [49]. Recently, the opioid crisis has stimulated intense collaboration, including shared syndrome definitions and analytic case-finding tools among geographically scattered institutions using ESSENCE [50-52].

The ongoing adaptation of ESSENCE to meet the needs of the understaffed public health practice community has provided a means to share methods and information, although data are often not shareable. The common analytic platform has enabled an evolving user ecosystem of multiple working groups, and the US CDC currently hosts the NSSP Community of Practice, with subgroups including Syndrome Definition, Data Quality, and Technical Committees [53]. The Syndrome Definitions committee promotes the analysis of common queries among geographically scattered user sites and the US CDC [54], thus improving communication between local and national health monitors. Epidemiologists, system designers, and other stakeholders may find and share resources on committee websites and through the NSSP Knowledge Repository [30]. A primary example is the opioid overdose crisis, a noninfectious threat. In addition to analytics and visualizations to support the activities related to this crisis, developers and users have worked together to acquire additional data sources, such as emergency medical incidents, poison center calls, and death records, to determine the benefit of fusing information varying in specificity and timeliness into a common surveillance picture to better inform awareness and interventions.

Regarding data standards that are essential among the many NSSP stakeholders, Multimedia Appendix 2 refers to the reference tables and business logic to convert data field entries into categorical values from standard sources such as the Public Health Information Network Vocabulary Access and Distribution System and from evolving standards developed by NSSP Data Sharing and Syndrome Definitions workgroups.

## Discussion

### Principal Findings

In the *Results* section, the multiple applications of ESSENCE installations by health departments at various levels and the surveillance community initiatives enabled and expedited by the system illustrate the combined effect of the technical components described in the *Methods* section and developed over 20 years, driven by the major influence of public health users. The following sections describe the lessons learned, key innovations, and user-driven enhancements.

### Lessons Learned

Several principles have driven the success of ESSENCE since its origins in the late 1990s.

#### Versatility

Users have valued the configurability and adaptability of ESSENCE. Default categorization of complex data into syndromic groupings has always been valuable to users who are inexperienced or who do not have the time to formulate or validate their own categories for monitoring. Conversely, health departments with greater analysis capacity have long demanded surveillance systems that allow them to create their own categories to track, and ESSENCE customization with query-building features using both diagnosis codes and free text has grown along with the sophistication and broadening needs of health department users. Precomputed, canned analysis products are not found in ESSENCE. However, versatility presents challenges to database design and to the selection and adaptation of statistical analysis tools. Surveillance data evolve with institutional information systems and formats, coding practices, and epidemiological concerns. Typically, users cannot wait for several minutes for data retrieval and time-consuming model runs. Alerting algorithms applied prospectively to detect disparate events in a wide variety of data types cannot match the detection performance of models developed retrospectively using historical data sets labeled with target events for a particular syndrome. The ESSENCE algorithm baselines do not span years, not only for storage and computational reasons but also because for many users’ desired data types, stable data or any data are available only within the past year. Hence, ESSENCE alerting algorithms, adapted from published applications of models and control charts in health care settings [55-57], use rolling baselines of weeks rather than years.

#### Facilitating Communication

Multiple ESSENCE users have remarked that one of the system’s main benefits has been to facilitate communication with other divisions within a health department, with external local and federal agencies, and with care facilities that provide data and can benefit from the broader geographic perspective that a surveillance system enables. Hence, substantial ESSENCE development has occurred in response to user requests for custom analysis comparisons, visualizations, and report formats, allowing overburdened users to concentrate on the task of routine health monitoring. In situations where data sharing is precluded by county or state regulations, ESSENCE communication tools have enabled information sharing. The sharing and query-building tools in the visualization section have been increasingly used to enhance collaboration among NSSP subcommittees as well as individual users.

#### Multiple Analysis Modes

The applicability of individual analysis modes, such as univariate and multivariate time series monitoring, spatiotemporal cluster detection, and single case identification all depend on the nature and quality of available data. For example, the spatial scan statistic implemented in ESSENCE can avoid issues of jurisdictional boundaries, but only if data location fields are present and reliably represented in the data. In many data sources, the limitation of location fields to zip codes or postal codes restricts the geographic precision of clusters of interest. Health monitors generally require multiple methods to analyze population health data. A notable example is that ESSENCE users in multiple health departments have discovered unreported cases of reportable disease that traditional sentinel surveillance is mandated to communicate to public health. The various analysis modes of ESSENCE provide affordable and sometimes beneficial redundancy and safety net functionality.

The following list summarizes these lessons for developers of other systems:

- Accommodation for a variety of processing systems and data types, flow, and format.
- Features facilitating shareable ad hoc queries and reports to investigate novel concerns.
- A suite of analytic tools allowing multiple looks at available data, tools that provide prompt feedback and do not require years of data history.
- Provision of default views as well as tools to modify and arrange them as desired while avoiding canned or precomputed visualizations.
- Integrated collaboration features and user events to foster direct community involvement in system evolution.

### Innovation

The development of ESSENCE has produced novel features in areas of complex, disparate data management, analytical methods, and the enhancement of user reporting and collaboration, interrelated efforts to empower health monitors. The data management advances include architecture and data transfer capabilities to meet the needs of institutions with varying resources. Analytics advances require data quality examination methods and alerting algorithms appropriate for diverse data time series that meet rapid response needs and do not require more than a few months of data history. User experience enhancements include customizable visualization and reporting features that provide unique time- and resource-saving capabilities.

### User-Driven Enhancements

Recent ESSENCE projects have produced a variety of user capability enhancements. For sharing information within and across jurisdictions, web-based features allow users to share what they are doing within ESSENCE with peers and to see what others are querying and find interesting. Text analysis and visualizations facilitate the creation of ad hoc local free-text queries. These features provide correlation, trend, and association analytics to help the user determine what terms or phrases should or should not be included in queries.

Back-end tools and checks with visualizations allow the user to closely monitor the local ESSENCE system for data issues and irregularities. These administrative capabilities help managers and users maintain day-to-day system availability and improve the visibility of issues that may develop over time.

Recently added visualizations and cohort clustering analytic tools for longitudinal assessment allow users to determine categories of patients who use health care systems that provide data to ESSENCE. These tools can show patient-level usage trends to inform the allocation of health care resources in a community.

### Conclusions

Installations of ESSENCE have provided systems capable of meeting public health surveillance requirements at multiple levels of purview and jurisdiction. The data architecture runs on modular configurations of a variable number of servers, with the number contingent on the data volume, number of active users, and frequency of required analysis operations; hence, the ESSENCE data architecture accommodates the acquisition and transfer processes of small and very large health departments. Essential to the growth of ESSENCE has been the capability provided to the user community to drive system development to meet its evolving needs. Users are provided with multiple syndrome and subsyndrome categories but are not limited by them or by canned or precomputed visualizations. Tools are provided to build simple or arbitrarily complex queries and dashboards for routine monitoring. The rich set of analytic methods includes alerting algorithms and other tracking tools applicable to sparse or rich data streams, with adjustment for only the most common data issues. These tools require historical baseline data of at most a few months, both accommodating limited historical data and providing fast turnaround for understaffed public health monitors. Although these algorithms cannot be tuned to particular syndromic categories or health events as in retrospective studies, they are designed for a range of common data types and behaviors. Current data missingness and lateness indicators are also provided to help users assess the reliability of the visualizations and statistics that they view. Finally, ESSENCE provides a growing suite of communications capabilities for customizable reporting, sharing, and convenient download for additional analytics. These combined features have streamlined onboarding, promoted methods and information sharing among the public health community, and enabled the diverse, all-hazards applications described earlier.

## Appendix

Multimedia Appendix 1 The Electronic Surveillance System for the Early Notification of Community-Based Epidemics (ESSENCE) history highlights.

Multimedia Appendix 2 The Electronic Surveillance System for the Early Notification of Community-Based Epidemics (ESSENCE) data architecture, security, and preprocessing.

Multimedia Appendix 3 Principles and details of The Electronic Surveillance System for the Early Notification of Community-Based Epidemics (ESSENCE) alerting algorithms.

Multimedia Appendix 4 Visualization tools in the Electronic Surveillance System for the Early Notification of Community-Based Epidemics (ESSENCE).

## Abbreviations

CCP: Chief Complaint Processor
CDC: Centers for Disease Control and Prevention
DoD: Department of Defense
ED: emergency department
ESSENCE: Electronic Surveillance System for the Early Notification of Community-Based Epidemics
JHU/APL: Johns Hopkins University Applied Physics Laboratory
NSSP: National Syndromic Surveillance Program
OTC: over-the-counter
SAGES: Suite for Automated Global Electronic bioSurveillance
VA: Veterans Administration
WRAIR: Walter Reed Army Institute for Research
