# Supplementary material for: Electronic Surveillance System for the Early Notification of Community-Based Epidemics (ESSENCE): Overview, Components, and Public Health Applications
Source: JMIR Public Health Surveill. 2021 Jun 21;7(6):e26303. doi: 10.2196/26303 (PMC8277331; doi:10.2196/26303)
Supplement: Multimedia Appendix 1 [file publichealth_v7i6e26303_app1.doc]

## ESSENCE History Highlights

The following items present the general chronological development of ESSENCE. The Results section provides citations of numerous selected individual applications to a range of public heath threat types.

1997: Early research in hospital information management led to a request by Maryland’s Secretary of Health to work with the Maryland Department of Health and Mental Hygiene to develop an automated system to detect bioterrorist attacks.

1999: The precursor to the current ESSENCE system, the Maryland Disease Surveillance and Reporting System (MDSRS), was implemented to monitor for bioterrorism.

Subsequent collaboration with the Department of Defense Global Emerging Infection System led to DARPA’s multi-center BIOALIRT program enabling improvements in algorithmic detection and biosurveillance system architectures.

2001: ESSENCE moved from a dynamic web framework to the static pre-built visualizations to exploit commercial GIS technologies. Limitations on user capabilities forced a rapid return to the dynamic web framework with a database backend for ESSENCE II.

Soon after the terrorist attacks of September 11, ESSENCE II was transformed from a limited-use research project to a live operational civilian surveillance system in Maryland and for all global US military treatment facilities.

2003: In response to the need to monitor the health of deployed US troops in the Iraq War, ESSENCE III was developed and deployed in March 2003. This version featured novel data sources, customizable syndrome groupings, and the first incorporation of external detection algorithms. The ability to implement external analytic methods prompted and validated the addition of an Application Program Interface (API) to the analytics architecture.

2003: Global emergence of the Severe Acute Respiratory Syndrome (SARS) spurred the need to sharpen and narrow case definitions in place of general syndromes. This threat solidified the decision to include free-text chief complaint queries along with diagnosis codes in the aggregations of monitored counts and rates. Tracking of SARS highlighted the power of automated systems for situational awareness, a broader and far more frequently useful objective than novel event detection. Subsequent experience began the expansion of syndromic surveillance from monitoring broad infectious syndrome groups to ad hoc categories of greater clinical specificity and a broader variety of population health threats.

2004: Expansion: the DoD Joint Services Installation Pilot Project (JSIPP) and the Department of Homeland Security (DHS) BioWatch program proliferated ESSENCE instances across the US. A common goal of these efforts was surveillance for rare but catastrophic events such as bioterrorist attacks, for which evidence in historical data was lacking. The influx of new users and widespread user sentiment that shareable and explainable results are no less challenging and important than event detection drove enhancements in visualization flexibility and exportability.

2004: The states of Maryland and Virginia and the District of Columbia agreed to share data across state lines, and the “Aggregated National Capital Region” (ANCR) version of ESSENCE was created. The Enhanced Surveillance Operating Group (ESOG) of ANCR users was formed to help guide ESSENCE development. The close tie between users and developers became a foundation for future ESSENCE enhancements.

2006: Expanded usage of ESSENCE including patient data from hundreds of hospitals at the Florida Department of Health led to additions to analytics including “time of arrival” monitoring in which anomalous groups of emergency department (ED) arrivals at hourly intervals were detected and reported with adjustments for patient volume, time of day, and subsyndrome type.

2007: The high-profile mass gathering events of the Super Bowl and the 2009 Presidential Inauguration impelled testing of information-sharing, distinct from data-sharing, strategies including an InfoShare tool to enable timely sharing by ANCR users with national level authorities. This effort foreshadowed multiple events or threats in which local restrictions prevented ESSENCE user sites from sharing explicit data, but derived reports, aggregates, data-free query language, or just descriptions could be legally shared. Implementation of ESSENCE features to facilitate such sharing has continued since then.

2009: Collaboration of the ESSENCE team with DoD-GEIS clarified the need to transition the technology and lessons learned from ESSENCE to an open source version that would be easy to operate, cost-free, and deployable around the world. The Suite for Automated Global Electronic bioSurveillance (SAGES) toolkit was developed, including OpenESSENCE and ESSENCE Desktop editions. While based on the features and functionality of ESSENCE, tools within SAGES were developed specifically for use in low- and middle-income countries.

Working with the Veterans Affairs and DoD, the ESSENCE team investigated the benefits and obstacles of including elements of electronic medical records beyond the demographics and chief complaints. Working with multi-terabyte database sites prepared ESSENCE developers to deal with large datasets in every jurisdiction.

2013: In one of the first applications to an injury-related health threats, the Boston Marathon bombings led to an ESSENCE addition to monitor non-infectious health categories including Anxiety, Depression, Suicidal Tendencies and Hearing Loss. These additions helped track the health of the Boston communities in the aftermath of the attacks.

2014: CDC announced the transition of the National Syndromic Surveillance Program (NSSP) to ESSENCE for syndromic systems analytics, to be hosted on the cloud-based BioSense Platform.

2016: Non-syndromic alerting algorithms were added to ESSENCE for rare, nonmedical, or uncommonly frequent terms in hospital free-text chief complaints.

2017: The opioid overdose crisis led to new, sustained partnerships between ESSENCE users and other public health divisions, such as injury prevention, behavioral health, and drug abuse. Analytic methods were explored to combine multiple data types for improved situational awareness. The MyESSENCE feature was added to allow users to customize dashboards for concise daily monitoring.

2018: The NSSP Syndrome Definition Committee users began sharing combined free-text and diagnosis code-based definitions for inclusion in local ESSENCE sites. Advanced natural language processing methods such as word embeddings were developed and shared among CDC and local users

2019: Nationwide monitoring of outbreaks of lung injuries associated with the use of vaping products increased demand for and development of system features to assess current data quality, including availability and informative content of the records underlying ESSENCE data displays.

2020: Collaboration among NSSP ESSENCE user groups intensified with evolution and sharing of queries related to COVID-19 pandemic surveillance.
