# Supplementary material for: Electronic Surveillance System for the Early Notification of Community-Based Epidemics (ESSENCE): Overview, Components, and Public Health Applications
Source: JMIR Public Health Surveill. 2021 Jun 21;7(6):e26303. doi: 10.2196/26303 (PMC8277331; doi:10.2196/26303)
Supplement: Multimedia Appendix 2 [file publichealth_v7i6e26303_app2.doc]

**ESSENCE Data Architecture, Security, and Preprocessing**

### Architecture

The software architecture employed for ESSENCE is a three-tier web application with a presentation layer as a user frontend, a business layer for application of algorithms, and a backend for databases. This architecture runs on modular server configurations, with the number of servers contingent upon the data volume, number of active users, and frequency of required analysis operations. The most common configurations comprise three servers for smaller instances and five servers for larger ones. For systems with larger numbers of data sources and/or data volumes reaching billions of records, the architecture can support additional servers to spread the functional load of the processing. The backend databases are Microsoft SQL Server [relational database management system](https://en.wikipedia.org/wiki/Relational_database_management_system)s (RDMS). Database functions include an ingestion database layer that facilitates extract-transform-load (ETL) operations and performs deduplication and other data cleaning operations. Depending on the user site, the ETL operations are performed by Rhapsody,[1] Mirth,[2] or locally developed scripts to populate the database, and then ESSENCE’s Groovy-based data flow management system [3] controls data flow and business logic to transfer data from ingestion to detection to web databases.

A detection database layer holds data and manages cube tables for fast algorithm access and execution. Java-coded algorithms access the data and cubes for efficient signal detection on the detection database to separate algorithm processing from user query management. The web database layer expedites rapid formation and display of interactive screens for visualization and communication. Web applications encoded in Java and JavaScript utilize this database via a Tomcat web application server.[4] For display purposes, mapping and other geographic information system (GIS) operations employ the open source tool GeoServer.[5] Users can access the web application through standard web application displays or via a web service API layer for direct access to ESSENCE data and functionality.

### Data Security

All automated data transfers occur over a secure virtual private networks (VPNs), e.g. via secure file transport protocol (SFTP); or over VPN tunnels; or by web transfer from a secure website, e.g. NPDS and weather. The use of web application access control allows limited, hierarchical access rights for every user by data source, by fields within a data source (e.g. geography or jurisdiction, syndrome), and by website function (e.g. data details, time series, alerts). These access privileges are customizable in the sense that one user site may control access by health district, others by county or by neighborhood. Some sites build aggregated datasets that meet the monitoring needs of users with limited access rights. Weather and other data sources with no privacy concerns may be made available to all users.

### Data Preprocessing and Quality Management

Multiple, data-dependent preprocessing steps include deduplication procedures, formation of syndrome fields, calculation of distances, and deriving additional fields and flags based on jurisdictional business rules and logic.

Procedures for managing data quality issues such as deduplication, temporary dropouts of data feeds, data field value validation, and management of free-text or pick-list entries are incorporated in ESSENCE standard business rules. By these rules, ESSENCE does not update records in place but employs a delete-and-replace approach that has proven faster. For data with the functionality enabled, a history system integrates all prior instances of each record to produce a single master record with the relevant fields for each encounter. An extensive set of reference tables and business logic allows conversion of field entries such as patient age, race, ethnicity, and vitals measurement such as temperature to categorical values from standard sources such as the Public Health Information Network Vocabulary Access and Distribution System (PHIN VADS). A “region” data field is used for general spatial aggregation of patient records and is most often employed to combine count data from sets of postal codes to approximate county-level counts when the county field is unavailable. Beyond these features and conventions, ESSENCE includes a substantial website section with guidance and analysis tools dedicated to helping users manage the quality of their data.

# References
