# Supplementary material for: Electronic Surveillance System for the Early Notification of Community-Based Epidemics (ESSENCE): Overview, Components, and Public Health Applications
Source: JMIR Public Health Surveill. 2021 Jun 21;7(6):e26303. doi: 10.2196/26303 (PMC8277331; doi:10.2196/26303)
Supplement: Multimedia Appendix 3 [file publichealth_v7i6e26303_app3.doc]

## Principles and Details of ESSENCE Alerting Algorithms

The following principles were derived with users to guide method selection and to clarify interpretation of results:

General considerations:

- These methods are not intended to positively identify outbreaks without supporting evidence. Their purpose is to direct the attention of a limited monitoring staff with increasingly complex data streams to data features that merit further investigation. They have also been useful for corroboration of clinical suspicions, rumor control, tracking of known or suspected outbreaks, monitoring of special events and health effects of severe weather, and other locally important aspects of situational awareness. Successful users value these methods more for the latter purposes and do not base public health responses solely on algorithm alerts.
- These algorithms are one-sided tests that monitor only for unusually high counts, not low ones. Low counts could result from a critical outbreak situation that prevents data reporting, but there are many more common reasons for low counts (such as unscheduled closings or system problems), so the algorithms do not test for abnormally low counts.
- In addition to data- and disease-specific considerations below, algorithm selection was also driven by system considerations. Users need to monitor many types of data rapidly. External covariates such as climate data or clinic schedules may not be available for prompt analysis. Many methods in the literature, armed with retrospective data of a certain type, depend on analysis of substantial history. Day-to-day users, often with only a small fraction of time available for monitoring, will not wait several minutes for each query. In the absence of data history and data-specific analysis time for each stream, ESSENCE methods have been adapted from the literature and engineered to system requirements.
- If the time series monitored by algorithms represent many combinations of clinical groupings, age groups, and geographic regions, excessive alerting may occur simply because of the number of tests applied. The Summary Alert method was implemented to limit such excessive alerting. This method is based on control of the false discovery rate, i.e. the expected ratio of false alerts to the total alert count, and its statistical implementation in ESSENCE is detailed in the Summary Alerts section below. Aside from analytic methods to control alerting, default alert lists should be limited to results from those time series of concern to the user, either by system design or by active specification by the user. For example, one method of reducing the default alert list is to restrict algorithms to all-age time series groupings. Depending on the scope of the user’s responsibility, the alert list may also be restricted according to both epidemiological interest and the resources available for investigation. For example, a monitor of a national-level system with algorithms applied to many facilities may be interested only in alerts with at least 5-10 cases. In circumstances of heightened concern, these restrictions can be relaxed, or the user can use ESSENCE advanced querying methods to apply algorithms to age groups and/or subsyndromes.

**Univariate temporal alerting algorithms**

The default temporal algorithm in ESSENCE is an automated selection between data modeling and control-chart-based algorithms, resorting to a simplistic Poisson distribution-based method if only a few days of recent data are available. The modeling method is adaptive multiple regression, while the control chart-like approach is an adaptive exponentially weighted moving average (EWMA) algorithm. The primary regression and EWMA methods are first discussed separately.

Each description below gives a method category, purposes of the method, a brief technical description, key benefits, limitations, and literature sources.

#### Algorithm: Linear Regression

Categorization: Adaptive Multiple Regression Model

Purposes: This model is an adaptive regression model applied to remove the systematic behavior often seen in time series of daily, syndromic, clinical visit counts and in other surveillance data. The reason for removing these common effects is to avoid bias in identifying unusual behavior. For example, there is a customary jump in visits on Mondays because many clinics resume normal hours, and this expected jump should not automatically increase the possibility of an alarm [1, 2]. Similarly, alarms should be possible on weekends even though visit counts drop off from weekday levels.

Technical Details: This adaptive, multiple, least-squares regression algorithm contains terms to account for linear trends, day-of-week effects, and holidays. Multipliers for these terms are calculated using 4 weeks of recent counts as a training period. This training period is separated from the date of the test data by a 2-day buffer intended to keep early outbreak effects from contaminating the training. Extreme data values in the training period are reduced to reasonable values in order to avoid exaggerated predictions. This outlier correction for model inference avoids loss of sensitivity in the weeks after either data problems or true outbreaks. The regression multipliers are recomputed each day for calculation of a predicted count based on the expected data trends. The algorithm then subtracts this prediction from the observed visit count, scales the excess by the standard error of regression, and applies a statistical hypothesis test to determine whether to signal an alert. The test is a Student’s t distribution at significance levels of 1% for red alerts and 5% for yellow alerts, with the number of degrees of freedom determined by the number of regression covariates and the baseline length [3]. Covariates and training intervals were chosen to obtain maximum sensivity for detection of injected signals at manageable background alert rates.

Benefits: The main benefit is avoiding alerting bias resulting from expected data trends. The length for the training baseline is critical. Based on performance comparisons among multiple baseline lengths, it was chosen to be short and recent enough to capture seasonal time series behavior but long enough to smooth out daily fluctuations. Separate multipliers are updated so that a data source with regular but unusual patterns such as high weekend counts will be modeled correctly. While a better fit may often be obtained with a more complex model for a given data stream with a certain syndromic filter for a certain subregion and analysis of sufficient data history, the current regression approach is relatively robust across time series employed in ESSENCE.

Limitations: If this algorithm is applied to a data series without the baseline weekly and seasonal behavior, the model will not explain the data well, and the detection sensitivity and specificity will be decreased. The automated switch in the default method is applied for this reason. There is no claim of optimal modeling for a given time series. This general-use implementation does not assume the availability of a denominator variable such as the total visit count that can be used to adjust the counts to emulate series of rates rather than counts. This adjustment has been implemented in particular versions of ESSENCE and in past versions of Biosense[4, 5] and could be added as an option.

#### Algorithm:Adaptive Exponentially Weighted Moving Average (EWMA)

Categorization: Adaptive Control Chart

Purposes: This algorithm is appropriate for daily counts that do not have the characteristic features modeled in the regression algorithm. It is more applicable for Emergency Department data from certain hospital groups and for time series with small counts (daily median below 10) because of the limited case definition or chosen geographic region.[6]

Technical Details: This algorithm compares a weighted average of the most recent visit counts to a baseline expectation. For the weighted average to be tested, an exponential weighting gives the most influence to the most recent observations. Two weightings are applied: the first gives negligible weight to observations over 3 days old and is designed to detect sudden events where most outbreak cases affect data within a few days. The second weighting distributes influence further over the past week for sensitivity to more gradual outbreaks. These weightings emulate a dual strategy published for the hospital setting. [7] The monitored weighted averages are the S_k_ given by:

S_k_ = ωS_k-1_ + (1- ω) X_k_,

for a constant smoothing coefficient ω with 0 < ω < 1 and X_k_ as the successive data counts, with X_0_ = 0 and S_0_ = half the alerting threshold for prompt sensitivity. (Occasionally a useful starting value for X_0_ is known, but restarts may occur for many reasons, so the conservative initialization to 0 is used.) For separate monitoring of sudden and gradual events, smoothing coefficients ω = 0.9 and 0.4 are used. For both weighted averages, the 4-week baseline mean is subtracted, with a 2-day buffer period to separate the baseline from the counts being tested. The rationale for the baseline length was the same as described above for the regression method above. The test statistic is then (S_k_ - μk) / σ_k_, where μ_k_ and σ_k_ are baseline mean and standard deviation. As in the regression method, the hypothesis applied to determine alerting is a Student’s t distribution at significance levels of 1% for red alerts and 5% for yellow alerts. The number of degrees of freedom assumed for this distribution is the baseline length + 1. This EWMA implementation is designed for any series that does not fit the characteristic trends, so a couple of safeguards are included. A “zero-filtration” algorithm is implemented for rapid adjustment to and recovery from data dropouts and catch-ups. When counts are sparse but not uniformly zero, a Poisson-based adjustment is added to the standard deviation scale factor to avoid excessive alerts. [3]

Benefits: This method gives sensitivity to both sudden and gradual outbreaks and has demonstrated prompt alerting capability. It is less susceptible than the Early Aberration Reporting System (EARS) methods C1, C2, and C3 to trends and to day-of-week effects. The added recovery features handle the most common problems in the data acquisition chain. Alerting is indirectly adjusted for the data distribution via the standardized residual test statistic, which provides a safeguard against excessive alerting when counts are small.

Limitations: This algorithm applied to pure daily counts does not control for expected trends or cyclic effects as in the regression method.

#### Algorithm: Poisson/Regression/EWMA (default)

Categorization: Automated switch between data model and control chart

Purposes: Many researchers and developers have applied complex statistical models to surveillance data for prediction and detection. However, the predictive capability of a model varies according to the specific data stream and how it is filtered and aggregated. This capability may also be affected by data behavior changes that result from seasonal variations, population shifts, and changes in the informatics. To account for such day-to-day changes, ESSENCE automatically monitors its predictive capability of its regression model each day. When this test fails, indicating that the model is not helpful for explaining the data, the system switches to the EWMA adaptation described above. The result is that the regression model is usually applied for the common respiratory and gastrointestinal syndrome classifications applied to county-level data, but EWMA is more commonly applied to rare syndrome data. For situations where less than a week of recent baseline data exists, a simple Poisson detector is applied. Such situations include new start-ups and more common restarts after long (several-week) intervals of missing data.

Technical Details: Details for the separate regression and EWMA methods are given in the preceding pages. The adjusted R^2^ coefficient for the regression is tested each day. This coefficient does not give the quality of regression but is employed here specifically as a measure of daily predictive capability using an empirically derived threshold criterion. When the data pass this test, the model is assumed to have explanatory value, and the regression algorithm is applied. When the data fail this test, the EWMA algorithm is applied. The Poisson distribution test is applied when less than a week (3-6 days) of recent data is available. A Poisson distribution is assumed with mean and variance equal to the mean of the recent counts. An alert is issued if the current count exceeds this mean and if probability that the current count was drawn from this distribution is less than 1% (red alert) or 5% (yellow alert). Practical safeguards for the composite method are as described in the regression and EWMA sections above.[3]

Benefits: This algorithm is the default because it is designed to avoid mismatching the method to the data. The regression model accounts for the expected data trends when they are seen in the baseline. When they are absent because of the case definition used to filter the data, because of the size of the monitored region, or because of data problems, alerting is based on the EWMA algorithm.

Limitations: The goodness-of-fit test occasionally misclassifies the data. The test is set to err toward the more conservative EWMA to avoid misfitting the data model.

#### Algorithm: C1, C2, and C3

Categorization: Adaptive Control Chart

Purposes: To purpose is to detect general data aberrations. Algorithms C1, C2, and C3 of the Early Aberration Reporting System (EARS) developed at the Centers for Disease Control and Prevention are used in many U.S. states and in numerous foreign countries.[8] They are included in the ESSENCE suite because of their wide application. While they lack many of the features described above, their simplicity has both benefits and limitations.

Technical Details: The C1 algorithm subtracts the daily count from the mean of a moving baseline ending the previous day. In effect, it then divides this difference by the standard deviation of counts in that baseline. If the result exceeds 3, indicating an increase above the mean of more than 3 standard deviations, an alert is issued. The C2 algorithm does the same calculation but imposes a 2-day buffer between the test day and the baseline. The C3 algorithm is a more sensitive version of C2 that adds the values from the 2 previous days if they do not exceed the threshold. All three algorithms use the same criterion of an increase of at least 3 baseline standard deviations above the sliding baseline mean. An important implementation detail is that ESSENCE does not use the standard 7-day baseline because substantial experience has shown that for many time series, such a short baseline gives an unstable statistic that can lead to a loss of confidence in the results. The implemented baseline is 28 days as in the EWMA and regression methods.[5] There are no other changes to the standard EARS methods, including retention of the flat 3-standard-deviation threshold regardless of the data stream.

Benefits: The methods are easy to understand and widely known.[9-11]

Limitations: Like the EWMA, the methods take no account of systematic data behavior such as day-of-week effects or seasonal trends. C3 is the only one of these methods with sensitivity to gradual outbreak effects, but it is known to produce high alarm rates. For all three methods, threshold data values for alerting may fluctuate noticeably from day to day.

### Summary Alerts--adjustment for multiple testing

Categorization: False Discovery Rate processing of multiple alerts

Purpose: The *parallel monitoring problem* is the monitoring of multiple separate time series representing different physical locations, such as counties or treatment facilities, possibly stratified by other covariates such as syndrome type or age group. The purpose of the Summary Alert Algorithm is to maintain sensitivity while limiting the number of alerts that arise from testing the numerous resulting time series.

Multiple testing can lead to uncontrolled alert rates as the number of data streams increases. For example, suppose that a hypothesis test is conducted on a time series of daily diagnoses of influenza-like illness. In a one-sided test, this test results in a statistic whose value in some distribution yields a probability p that the current count is as large as observed. For a desired Type I error probability of ***α***, the probability is then (1- *α*) that an alert will not occur in the distribution assumed for background data. Thus, for the parallel monitoring problem of interest here, if such tests are applied to N independent data streams, the probability that no background alerts occur is (1- *α*)*^N^*, which decreases quickly for practical error rates ***α. ***For a single-test error rate of ***α ***= 0.05, for example, the probability of at least one background alert exceeds 0.5 if more than 13 independent tests are applied.

Technical Details: For N tests, where N is the number of combinations of region, syndrome, age group, and any other covariates affecting the number of tests, let *P*_(1)_,…, *P*(*_N_*) be the *p*-values sorted in ascending order, an ordering that puts the smallest and most significant p-value first. The Summary Alert method applies the Simes-Seeger-Eklund criterion to reject the combined null hypothesis of no anomaly for any series.[12] The null hypothesis is rejected if for some *j**, *j** = 1,..,N, *P*(*_j*_*)  <  *j*α*  / *N*.   To interpret this condition, note that for the most significant p-value, an alert requires that *P*(*_1_*)  <  *α/N, *the strict Bonferroni bound. If *α*=0.01 and N=50, then the condition becomes *P*(*_1_*)  < 0.0002*.* For the least significant p-value, the condition is simply *P*(*_N_*)  <  *α,* highly unlikely for the weakest result.

If this condition is satisfied for any j*, then test results are considered alerts for all j < j*.[13] The Summary Alert is implemented at two levels, FDR and FDR-Major. For the FDR level applied to N time series, the implementation is as above. For a more liberal option appropriate for certain syndromes or scenarios, FDR-Major applies the condition to two sets of N/2 time series.

Benefits: In defining the false discovery rate as the expected ratio of false alerts to the total alert count, Benjamini and Hochberg showed that the Simes-Seeger-Eklund criterion gives an overall error rate of *α* if the N time series tested are statistically independent.[14] Overall, this criterion avoids the excess alerting resulting from using the nominal threshold *α* for all data streams and also avoids the loss of sensitivity from using only the Bonferroni bound *α/N*.

Limitations: If one of the p-values crosses the adjusted threshold, it is not obvious for epidemiological or other reasons which tests to consider anomalous. Most users have followed the natural procedure described by Simes to consider all p-values less than *P*(*_j*_*) as individual alerts. Another limitation is that in general the time series are not statistically independent. For situations where dependence is known, Hommel recommended the condition *P*(*_j_*)  <  *j* ∙ i  /  *C* ∙ *N*, where *C* = Σ 1/*j*. In ESSENCE applications where many groups of time series may be requested and dependence can change, the above condition with C=1 is applied.

### Spatial cluster determination

Categorization: Spatial Scan Statistics

Purpose: A problem with sophisticated temporal detectors is choosing the appropriate size and location of the collection region for time series counts. If this region is too small or mislocated, cases may be missed and the baseline data may not have enough structure, but if the region is too large, the scale and variability of the large-scale time series may reduce sensitivity by masking clusters of interest. We apply spatiotemporal scan statistics in an attempt to promptly localize public health problems. For ESSENCE, JHU/APL built and implemented a Java version of the algorithm implemented in the SaTScan software of Martin Kulldorff originally developed for spatial surveillance of cancer and subsequently used and enhanced for many types of hotspot detection.[15]

Technical Details: The null hypothesis is that the set of data subregions (often zip codes) in the recent time interval tested forms a random sample from an expected spatial distribution of cases. The expected distribution is not uniform over subregions but reflects a “customary” spatial case spread that reflects urban/suburban case ratios or other factors. ESSENCE implementation calculates the expected spatial distribution using recent case counts from a sliding baseline interval. In effect, the code is similar to a common application of SaTScan, the space-time permutation scan statistic, restricted to test cases from only the most recent time interval and assuming circular clusters.[16]

As in SaTScan, the method calculates a test statistic for each candidate cluster. The test statistic in the ESSENCE implementation is Kulldorff’s Poisson log likelihood ratio. The set of candidate clusters is generated by scanning over a set of cluster center locations, often taken as centroids of all zip codes in the dataset, and considering all circles within a maximum radius of each center, where the number of circles is limited by the number of data subregions within each radius. The maximum test statistic over these candidates is then tested for significance.

Statistical significance inference does not depend on a theoretical distribution but on repeated trials on simulated datasets randomly drawn using the baseline distribution. For each such trial, the algorithm uses the same scanning procedure to derive a trial maximum.

For assessing the significance of the maximum test statistic over all observed clusters, the ESSENCE code uses the Gumbel distribution method.[17] The code collects 99 trial maxima, fits a Gumbel distribution to these values, and uses the fitted distribution to assign a p-value to the test statistics of clusters found in the original data. The observed cluster with the maximum test statistic is considered significant if its p-value is below a predetermined threshold, often set to 0.01. This threshold criterion can yield multiple significant clusters in a given run if more than one candidate cluster yields a test statistic whose p-value is below the threshold. For each significant case cluster, the system shows the location, extent, and degree of significance using the GIS software.

Benefits: The ESSENCE Java implementation inherits features that have popularized SaTScan. Potential clusters of interest are localized without bias regarding the center or extent of the cluster as well as the spatial resolution of the data allows. As noted in Kulldorff, Heffernan, et al., the empirical significance testing with many repeated trials takes “into account the multiple testing stemming from the many potential cluster locations and sizes evaluated.”[16]

Limitations: The most important limitation, applicable also to SaTScan and to all other spatial or space-time cluster detection methods, is that the usefulness of the method strongly depends on the reliability of the expected spatial distribution. The use of census-based distributions, insurance eligibility lists, regression models, and other means have been used to derive the expected distribution. The method implemented in ESSENCE infers this distribution from recent data separated from the test date(s) by a 2-day buffer.

Evaluation of statistically significant clusters for epidemiological significance is a nontrivial task which may be exacerbated if the number of significant clusters is misleading or excessive because the expected distribution is unrepresentative or because investigation resources are insufficient.

The use of this popular approach for prospective use has been criticized despite numerous applications and published real-life successes,[18] and the ESSENCE implementation lacks the prospective adjustment in SaTScan attempting to manage cluster rates for multiple successive runs. The ESSENCE implementation also does not support elliptical cluster shapes, simultaneous clustering of multiple data sources, or test statistics other than the Poisson log likelihood ratio. The user with a sufficiently detailed dataset and an application that requires these extended SaTScan features should be aware of these limitations.

### Time-of-arrival aberration detection

Categorization: Multiple Automated Hypothesis tests

Purpose: This algorithmic approach was implemented to find and display unusual clusters of syndromically related emergency department visits by patients arriving for care within a short time interval.

Technical Details: Patient visit counts are tabulated by cells, with one cell for each hospital/time-interval/sub-syndrome combination. See Figure S1.


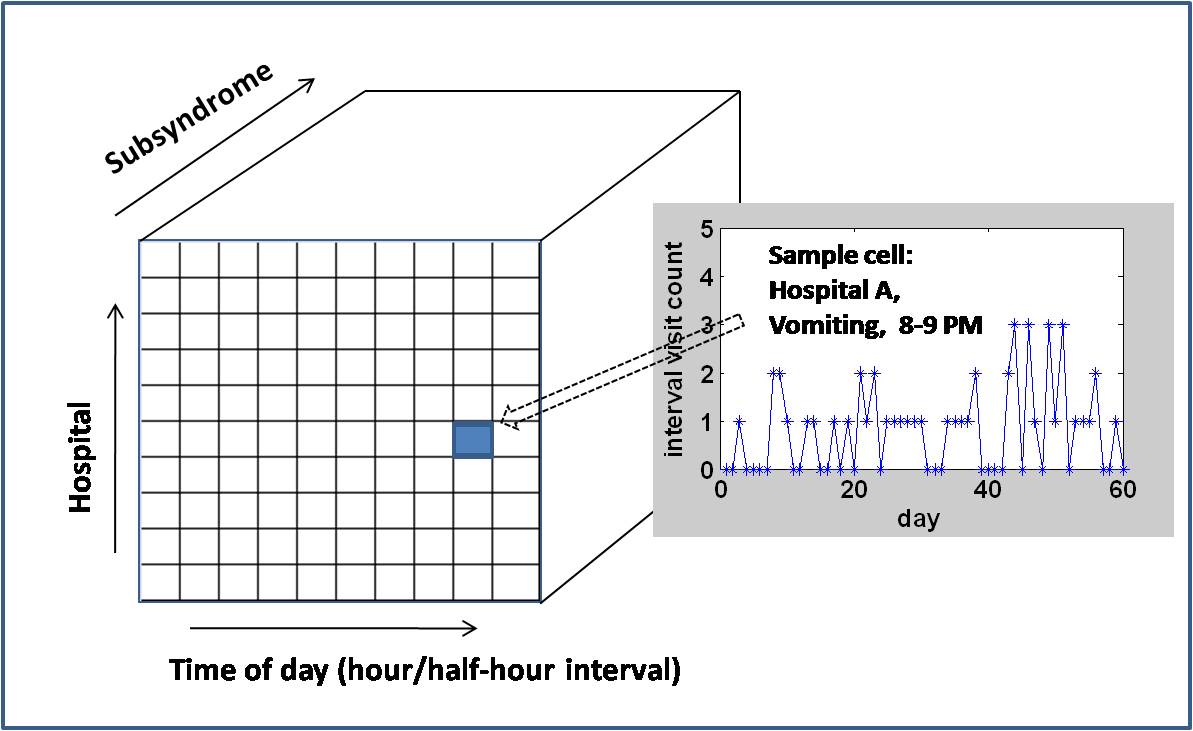


**Figure S1: Schematic of time-of-arrival filtering and anomaly detection process to identify unusual sets of visits in 30- or 60-minute intervals, for multiple subsyndromes of emergency department data**

- For the visit counts in each cell, a Poisson or negative binomial test is chosen using the last 60 days of visit counts for that cell. The Poisson distribution is used unless the count variance exceeds the mean by a factor of 1.1 or greater, and then the time series is considered overdispersed. This situation occurs for relatively few cells, generally corresponding to the more common (sub)syndromes for the largest hospitals at the busiest times when most alerts would be generated. For this situation, a negative binomial distribution is assumed. Parameter settings and distribution types were derived from testing on several years of patient visit records from a variety of large and small hospitals.
- Once the distribution is chosen, parameters for each cell are calculated from the 60-day baseline. For each cell, an alert is then flagged if the current count exceeds the upper limit threshold for the chosen distribution based on a preselected p-value.
- Based on empirical results using 12 years of data from 134 hospital EDs from a large state with labeled events, a threshold p-value of p* = 1E-4 (0.0001) was chosen.
- Time intervals for the cells are 30 min., 60 min. beginning on the hour, and 60 min. beginning on the half hour, again a result of empirical testing.
- Practical overrides are implemented based on observed cell counts. At least three observed cases are required for an alert. This minimum may be increased for more common syndromes. Mandatory alerts may also be implemented for certain subsyndrome/count combinations, such as subsyndromes for severe illness, regardless of the hypothesis test.

Benefits: In validation testing to monitor visit clusters for 51 subsyndromes for 134 hospitals at the time intervals above with the chosen p-value threshold, alert rates were consistently manageable and found all known clusters from a small historical collection of events except for two groups of 3-4 visits at very busy times. The alert burden was still manageable at the county level when anomalous clusters for all hospitals within each county were combined. The simplicity of this approach allows multiple daily runs and adaptation to new improvised subsyndromes with rapid system response without impact on routine processing.

Limitations: The hypothesis tests include no direct modeling of seasonality or other systematic data behavior. They were implemented to enable county-level processing, and validation was conducted on a 12-year historical dataset from one state. Expanding the computational load to include much larger sets of hospitals or syndrome groups with limited investigation capability may require recalibration (p-value threshold, minimum alert counts) or an alternate approach to retain sensitivity with manageable alerting.

### Identification of term-based free-text anomalies

Categorization: Hypothesis test for excessive occurrence of selected chief complaint terms

Purpose: This algorithm was implemented to point out unusual distributions of chief complaint terms of interest without dependence on syndrome definitions.

Technical Details: For each term in any chief complaint in the most recent time interval, the number of chief complaints containing that term in this interval is tested relative to the number of chief complaints containing same term in a much larger sliding baseline interval, assumed to be representative of customary data.

- The algorithm forms 2x2 contingency tables whose entries are:

A=number of recent chief complaints with the term,

B=number of recent chief complaints without the term,

C=number of baseline chief complaints with the term,

D=number of baseline chief complaints without the term

- For many such tables with small cell counts, Fisher’s Exact Test is applied to alert when the probability that count of the term of interest is ≥ A, given the contingency table’s marginal totals, is below a critical p-value.
- If the smaller of B and C is larger than 1000, then a chi-square test is applied to the same contingency table with negligible loss of accuracy. The critical threshold is then applied to half the resultant (two-sided) p-value to determine anomalous terms.
- Results are shown only for candidate terms that have not been previously eliminated because they are stopwords with no informational content (such as “the”, “if”, “all”) or because users have previously added them to a list of terms to be ignored (such as “patient”, “complaint”, “test”).
- Conventions adopted from empirical test results and also to avoid impacting ESSENCE processing are: The test interval is 24 hours, the baseline interval is 30 days, a buffer of 7 days is implemented between test interval and baseline, and the threshold p-value is set at p* = 10^-5^ (0.00001).

Benefits: With the above p-value threshold and settings, this method detects as few as three instances of unusual terms (place names, rare signs/symptoms) and unusual concentrations of interesting common terms while averaging from 0-4 anomalous terms per day over small and large hospitals. Inspecting chief complaints containing each of a small number of terms each day and disqualifying some terms from further consideration is a manageable task that can uncover clusters of visits that could be missed by syndromic methods. In testing with historic data, chief complaints containing anomalous terms found with the strict p-value threshold adopted have included small clusters of visits resulting from documented events of food poisoning and heat-related illness. This analysis has also found new abbreviations used by hospitals in their chief complaints. These new abbreviations can then be added to the Chief Complaint Processor to improve syndrome and subsyndrome categorization.

Limitations: While the simplicity of this method avoids impact on daily ESSENCE processing or user investigation of large collections of distributed data streams, the method has no means to interpret anomalous terms, identify phrases with multiple terms, or distinguish topics of concern in chief complaints that may not share specific terms. The only preprocessing of free-text terms is the application of ESSENCE Chief Complaint Processor spell-checking and abbreviation/acronym expansion. Interpretation of the anomalies requires a human-in-the-loop, both to evaluate anomalous terms for investigation and to rule-out specific terms from future anomalies. The method is subject to effects of changing terminology (street drug names, triage vocabulary and abbreviations), and the user should be aware of current perceived health threats and corresponding emergency/urgent care language.
