# Supplementary material for: Electronic Surveillance System for the Early Notification of Community-Based Epidemics (ESSENCE): Overview, Components, and Public Health Applications
Source: JMIR Public Health Surveill. 2021 Jun 21;7(6):e26303. doi: 10.2196/26303 (PMC8277331; doi:10.2196/26303)
Supplement: Multimedia Appendix 4 [file publichealth_v7i6e26303_app4.doc]

**Visualization Tools in ESSENCE**

Both standard and user-customizable visualizations are available in ESSENCE. Highly configurable and interactive modes of data stratification and filtering, graphical and tabular customization, user preference management, and sharing features allow users to query data and view geographic representations, time series and data details pages, and reports. The following sections summarize key features. Screenshots of selected novel visualizations are shown below.

### Standard Visualizations

Following are the most commonly used ESSENCE visualizations:

- The Time Series View provides a graphical display of the temporal behavior of the data with the ability to stratify by specific parameters, view aggregated counts, and infuse data quality factors to improve understanding of data features.
- The Data Details Page provides line listings of individual records and pie/bar chart representations of query results.
- The Map View allows the user to view both data and alerts on a geographical display of the specified region.
- The Alert List provides a view of signals generated by the alerting algorithms. Each row of the list includes a link to the corresponding Time Series View or Data Details Page.

To facilitate selection of desired data by space, time, subpopulation, and clinical specificity, the Query Portal provides the user with tools for detailed design and management of simple or complex queries for routine or ad hoc monitoring. An online Query Wizard facilitates these processes as in Figure S1. For the desired analysis, the user chooses the data source, date range, time resolution (daily, weekly, monthly, quarterly, or yearly), temporal detection algorithm, and selections from a variety of component filters depending on the data source. For each data source, the user can define custom filters through configuration files that customize visualizations. Options include Free-Text, Reference List, Number Range, and Dates filters. Examples of query field options for filter creation are age group, geography system, syndrome or medical category of interest.

Details of the attributes available for selection, stratification, and filtering are:

- **Geography System:** Data can be viewed in many geography systems such as Region, Zip Code, Hospital, Region of the Hospital, Military Treatment Facility (MTF), School, or Store. Each geography system defines a system for geographically filtering your data. Regions are a generic term that defines the default geographic way to view data. Regions normally map to a set of zip codes that closely resemble a county or health district.
- **Medical Grouping System:** When viewing the data via the Query Portal, the user has the ability to choose between how the various data are presented. Data may be viewed by many grouping systems, including ESSENCE Syndrome, International Classification of Diseases (ICD) Code, Chief Complaint SubSyndrome, Chief Complaint, OTC Category for records of sales of over-the-counter remedies, or Call Center Guideline, depending on which data source is queried.
- **Syndrome Grouping:** The syndrome groupings used in the ESSENCE system vary depending on the medical grouping systems used and the needs of the user site. These groupings are used to filter data into medically similar sets. Examples of a syndrome grouping are: Respiratory, Rash, Cough/Cold, Sinus, Asthma, Chest Pain, etc. Each medical grouping system will have a set of syndromes or the ability to perform free text queries. A Syndrome Definitions user interface provides a stepwise mechanism for viewing rules that define a syndrome or subsyndrome. In addition to syndrome categories the user may also query based on a Chief Complaint Discharge Diagnosis (CCDD) field, a concatenation of the parsed chief complaint and discharge diagnosis. Filtering by CCDD category uses Structured Query Language (SQL) “Where” clauses to select records meeting user criteria. In general, this filtering takes the form of simple keyword matching with inclusion of wildcard matching and negation terms.
- **Detector:** The ESSENCE tools refer to alerting algorithms as *detector*s. For temporal alerting methods, users may choose any of the methods described in Additional File 1.


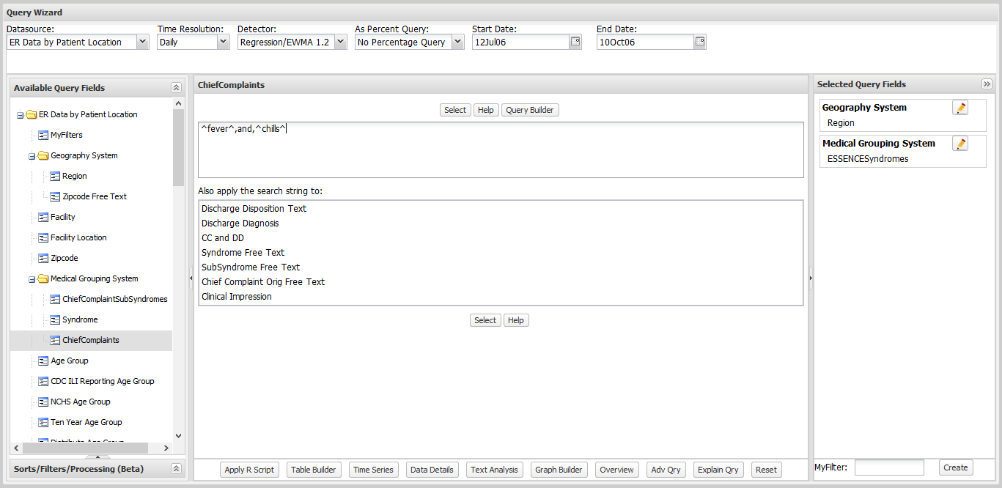


**Figure S1: Screenshot of the The ESSENCE Query Wizard window for custom, shareable queries of selected features of any available data source, enabling ad hoc syndrome formation**

For queries involving more complex data selection and free-text logic than the query wizard provides, ESSENCE provides an Advanced Query Tool, shown in Figure S2.


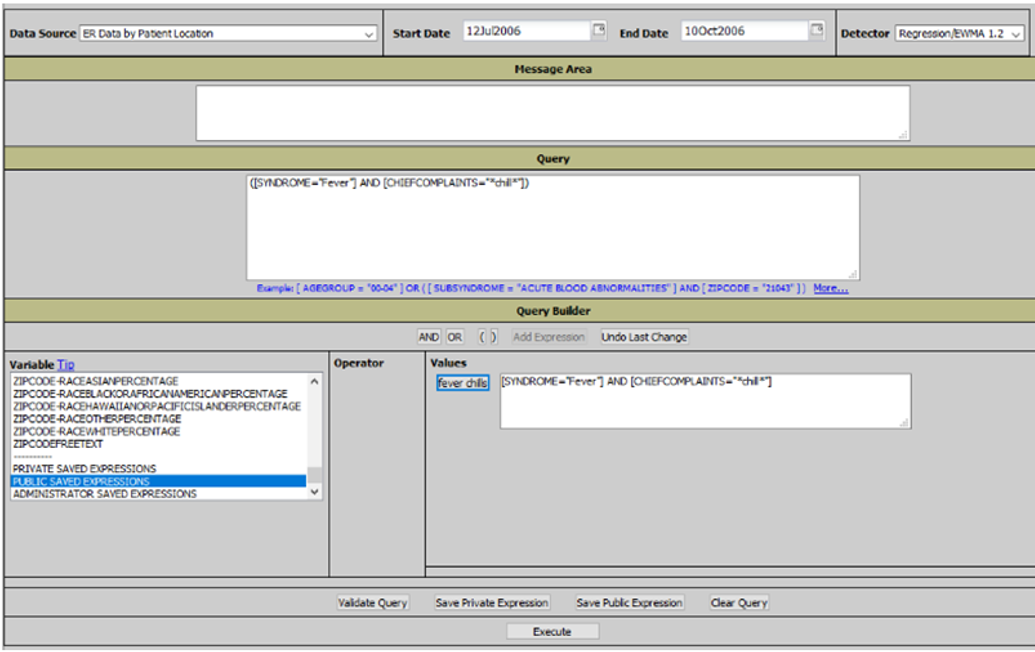


**Figure S2: Screenshot of the Advanced Query Tool in ESSENCE, allowing queries involving complex data selection and free-text logic, with features for automated logic validation and public or private sharing.**

Once a user has created a query, an action button is available to indicate the use and disposition of the query. Options include formation of time series and tables. Complex queries can be saved for reuse and for application to other appropriate data sources.

### User-Customizable Visualizations

In addition to the standard visualizations, ESSENCE offers additional views and analysis modes. Of these, the most commonly used are myESSENCE and myAlerts.

**myESSENCE**: The myESSENCE feature on the main ESSENCE menu facilitates creation of multiple customized dashboards of graphs, charts, tables, maps, and alerts. Users may create separate dashboards by using a widget interface to select, drag, and drop widgets. They may share each dashboard with other users by providing a copy to the dashboard or by providing a read-only copy over which they maintain control. Possible choices for the widgets are time series graphs, maps, and listings of data details. Parameters for each view, such as start and stop dates, are modifiable. These views may be arranged in 1-, 2-, or 3-column format. Figure S3 exemplifies the dashboard creation process.

The creating user gives each dashboard a text description and a note that is modifiable by creator or shared user. Once created, each dashboard appears as a separate tab on the myESSENCE webpage.

By default, each dashboard applies to data from the geographic regions selected for each graph when added to the dashboard. The creator or sharing users may change the region, and ESSENCE will change all views on the dashboard to reflect data from the new region. Users may revert to the default view by choosing “Original Query”.


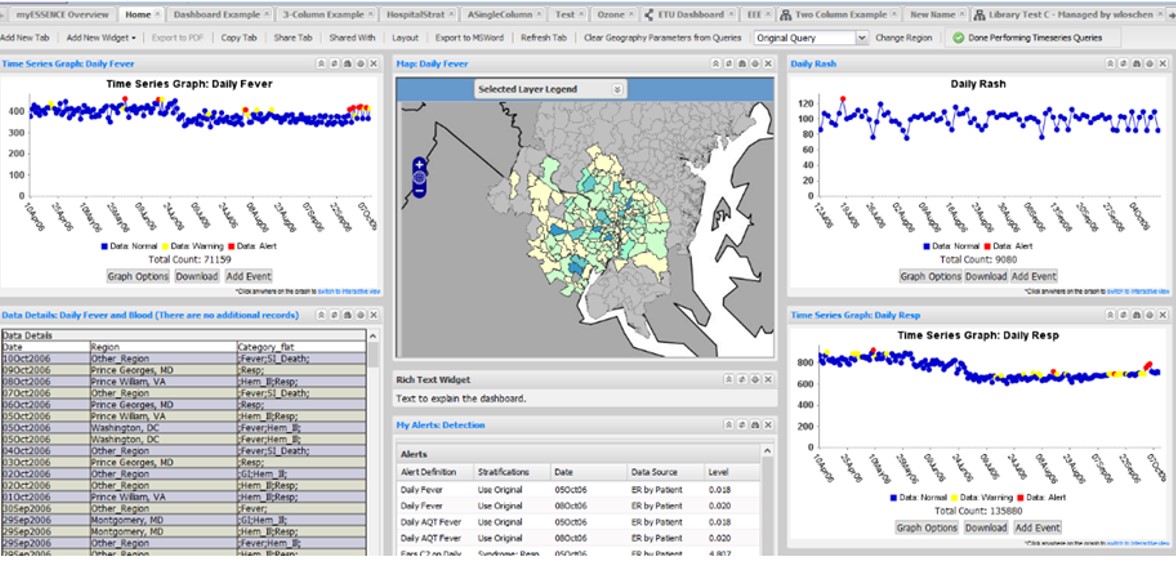


**Figure S3: Screenshot of customized dashboard Using MyESSENCE Feature, with plots, maps, charts, data details, and text selected and arranged by users for rapid daily situation estimation.**

***myAlerts***: The myAlerts feature enables users to set up automatic notifications of query results reflecting specific subpopulations, algorithms, and conditions. Two types of these notifications are available. Notifications triggered by receipt in ESSENCE of any single data record that satisfies a query definition are *Records of Interest* alerts. Notifications triggered by minimum count requirements or by algorithms detecting sets of records returned by a query are *Detector* alerts. Algorithms specified may be any of the methods described in Additional File 1. The user may also specify a minimum number of records requirement in addition to an alerting algorithm. Figure S4 shows the web page used to create this customized alerting feature. Alerts that require algorithm results that cross a designated statistical threshold may be restricted to require threshold crossings on m consecutive days, or on m of the last n consecutive days. The user may set up automatic email prompts for each customized alert and may choose automated sharing of selected alerts with other designated ESSENCE users.

Developers of ESSENCE have also created single-purpose views and tools for specific jurisdiction needs, including statistics tables and side-by-side graphs, as broadly applicable as possible to all data sources.


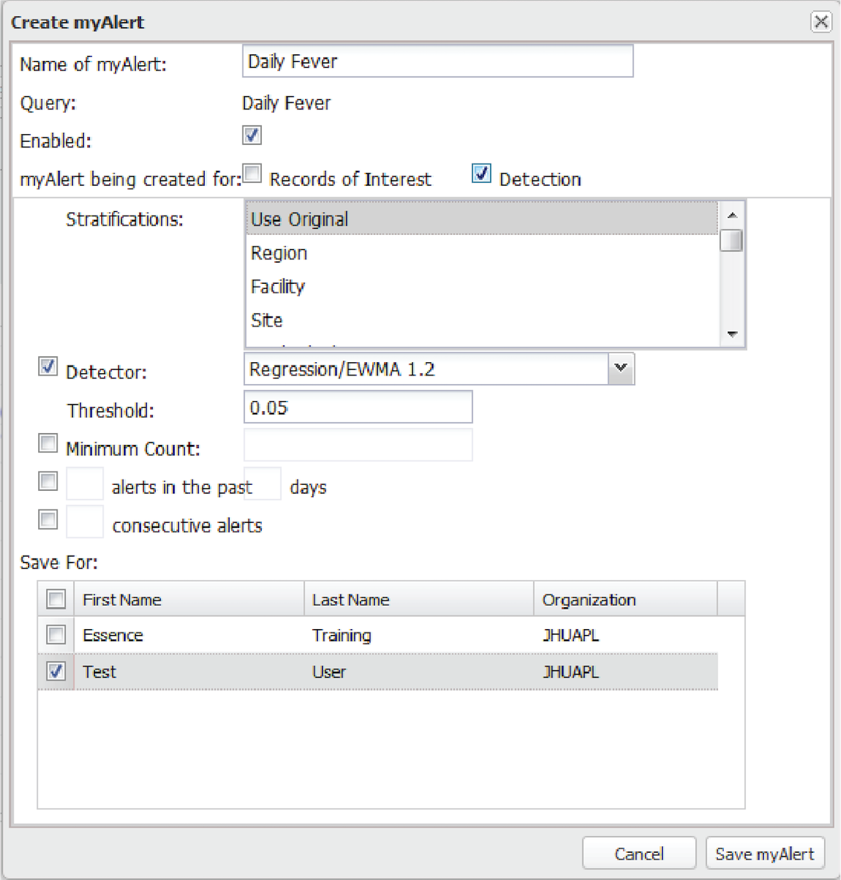


**Figure S4: Screenshot illustrating creation of MyAlert for customized anomaly detection, with multiple selectable alerting criteria for monitoring preferred data source features.**
